# Oxygen Tension Strongly Influences Metabolic Parameters and the Release of Interleukin-6 of Human Amniotic Mesenchymal Stromal Cells *In Vitro*

**DOI:** 10.1155/2018/9502451

**Published:** 2018-10-28

**Authors:** Asmita Banerjee, Andrea Lindenmair, Ralf Steinborn, Sergiu Dan Dumitrescu, Simone Hennerbichler, Andrey V. Kozlov, Heinz Redl, Susanne Wolbank, Adelheid Weidinger

**Affiliations:** ^1^Ludwig Boltzmann Institute for Experimental and Clinical Traumatology, AUVA Research Center, Donaueschingenstraße 13, 1200 Vienna, Austria; ^2^Austrian Cluster for Tissue Regeneration, Vienna, Austria; ^3^Ludwig Boltzmann Institute for Experimental and Clinical Traumatology, AUVA Research Center, Garnisonstraße 21, 4020 Linz, Austria; ^4^Genomics Core Facility, VetCore, University of Veterinary Medicine, Veterinaerplatz 1, 1210 Vienna, Austria; ^5^Red Cross Blood Transfusion Service for Upper Austria, Krankenhausstraße 7, 4017 Linz, Austria

## Abstract

The human amniotic membrane (hAM) has been used for tissue regeneration for over a century. *In vivo* (*in utero*), cells of the hAM are exposed to low oxygen tension (1–4% oxygen), while the hAM is usually cultured in atmospheric, meaning high, oxygen tension (20% oxygen). We tested the influence of oxygen tensions on mitochondrial and inflammatory parameters of human amniotic mesenchymal stromal cells (hAMSCs). Freshly isolated hAMSCs were incubated for 4 days at 5% and 20% oxygen. We found 20% oxygen to strongly increase mitochondrial oxidative phosphorylation, especially in placental amniotic cells. Oxygen tension did not impact levels of reactive oxygen species (ROS); however, placental amniotic cells showed lower levels of ROS, independent of oxygen tension. In contrast, the release of nitric oxide was independent of the amniotic region but dependent on oxygen tension. Furthermore, IL-6 was significantly increased at 20% oxygen. To conclude, short-time cultivation at 20% oxygen of freshly isolated hAMSCs induced significant changes in mitochondrial function and release of IL-6. Depending on the therapeutic purpose, cultivation conditions of the cells should be chosen carefully for providing the best possible quality of cell therapy.

## 1. Introduction

Oxygen is the element of highest electronegativity after fluorine, and even molecular oxygen is still a highly reactive and therefore, toxic molecule. Photosynthesis by organisms in early evolution led to the “oxygen explosion” in the earth's atmosphere 2.3 billion years ago, causing mass extinction of anaerobic species [1, 2]. By hypothesis, only the entering of an oxygen-utilizing prokaryote into a preeukaryote allowed surviving an oxygen-containing atmosphere [3]. This process of endosymbiosis, marking the birth of mitochondria, changed the course of evolution [3] (reviewed in [4]).

Tissue oxygen tension is a measure of the oxygen partial pressure in the interstitial (extravascular) space reflecting the balance between oxygen supply and demand (reviewed in [5]). Oxygen tension in cellular microenvironment can strongly influence cellular processes (reviewed in [6]). In the course of evolution, organisms developed numerous strategies to cope with the potentially toxic effects of oxygen, such as antioxidant systems and enzymes. Exposure to oxygen in tissues is therefore highly regulated by the level of vascularization (reviewed in [5]). Mammalian tissues have highly specific oxygen tensions which can range from 16% oxygen in alveolar air (reviewed in [5, 7]) down to almost anoxic calculated oxygen tension in the bone marrow hematopoietic compartment [8, 9]. Therefore, different cells of the body are exposed to different oxygen tensions. Adult stem cells, such as mesenchymal stem cells, neural stem cells, and hematopoietic stem cells, maintain their stem cell state in niches of very low oxygen tensions (reviewed in [10, 11]). An increasing number of publications have demonstrated energy supply via glycolysis in embryonic [12, 13] (reviewed in [14]) and mesenchymal stem cells [15–18] and even induced pluripotent stem cells (iPSCs) [19]. According to current models, stem cells remain quiescent in their specialized niche [20] (reviewed in [21, 22]) until external signals induce a metabolic shift. Stem cells then switch their metabolism from primarily glycolytic to oxidative phosphorylation and start to differentiate towards progenitor and precursor cells (reviewed in [23]). Therefore, it is now widely accepted that maintenance of a stem cell state requires maintenance of their highly specific microenvironment.

An attractive source of adult stem cells is the human amniotic membrane (hAM) [24]. This fetal membrane consists of an epithelial layer, formed by a monolayer of human amniotic epithelial cells (hAECs), and a collagen-rich mesenchymal layer, in which the human amniotic mesenchymal stromal cells (hAMSCs) are embedded. In clinics, the hAM has been used decellularized or denuded for tissue regeneration purposes for over a century [25, 26]. Transplanted hAM does not cause rejection reactions in the patient (reviewed in [27]), and furthermore, the hAM and the cells thereof have anti-inflammatory [28–36] and immunomodulatory properties [37–39]. Additionally, cells of the hAM can differentiate into cells of all three germ layers *in vitro* and *in vivo* [40–44]. Therefore, the use of cells of the hAM for tissue regeneration has moved into the focus of many research groups.

While common cell culture conditions derive originally from cultivations of chicken fibroblasts at 20% oxygen, other cells, such as stem cells, need a more specialized oxygen microenvironment. Changes in the oxygen microenvironment particularly affect mitochondria, also designated as the “main sink of oxygen” [45]. Oxygen, with its high standard redox potential, is the final electron acceptor in the mitochondrial electron transport chain for the generation of adenosine triphosphate (ATP) via oxidative phosphorylation. This metabolic process also releases superoxide, a reactive oxygen species (ROS), predominantly produced by mitochondrial complexes I and III [46, 47]. ROS, formerly considered as mere damaging byproducts, came recently into focus for their signalling function (reviewed in [48]). Therefore, it does not come as a surprise that mitochondrial function plays a critical role in maintaining stemness [49], orchestrates cell fate (reviewed in [23]), and also plays a critical role for tissue regeneration [50].

*In vivo*, cells of the hAM are exposed to low oxygen tension (1–4%; [51]) while *in vitro* cultivation or storage is usually performed at 20% oxygen. As changes in the microsurroundings of hAMSCs in culture may impact cellular processes, we tested the influence of low (5%) and high (20%) oxygen tensions on mitochondrial function of freshly isolated hAMSCs after 4 days in culture. As we found different mitochondrial activities in reflected and placental amnion biopsies in a former study [52], we separately investigated hAMSCs from placental (P-hAMSCs) and reflected amnion (RA-hAMSCs). Furthermore, as the anti-inflammatory properties of the hAM represent a potentially crucial function in a clinical transplantation situation, we also measured parameters linked to inflammation. The results of this study could support the possibility of specific selection and preparation of amniotic cells according to clinical requirements.

## 2. Material and Methods

### 2.1. Preparation of the Human Amniotic Membrane

Placentae were obtained from planned caesarean sections from healthy patients at full term. The patients had signed informed consent with approval of the local ethics committee, in accordance to the Declaration of Helsinki. Placentae were transported within 4 hours of delivery, in 500 mL Ringer solution. Placentae from caesarean sections of premature deliveries, emergency caesarean sections, and placentae with detached amniotic membranes were excluded from the study. The reflected and placental regions of the hAM were separated from each other as previously described [52].

### 2.2. Isolation of Human Amniotic Mesenchymal Stromal Cells

Isolation of hAMSCs was performed as previously described [33]. Briefly, reflected and placental amnions were cut into 2 × 2 cm pieces, incubated in 1 mg/mL collagenase solution, and shaken for 2 h at 37°C. Digestion was stopped with cold PBS, and the cell suspension was filtered through 100 *μ*m cell strainers and centrifuged at 4°C for 9 min at 400 g. The cell pellet was resuspended in DMEM, supplemented with 10% FCS, 2 mM L-glutamine, and 1% penicillin/streptomycin (medium and supplements from Sigma-Aldrich, USA). Cell yields of approximately 15 *×* 10^6^ cells for reflected amnion and 10 × 10^6^ cells for placental amnion were reached per donor, depending on the size of the hAM. Donors with more than 10% dead cells (staining positive for trypan blue) were excluded from the study.

### 2.3. Cultivation of Human Amniotic Mesenchymal Stromal Cells at 5% and 20% Oxygen

Freshly isolated hAMSCs of both amniotic regions, reflected and placental, were cultured for 4 days at 37°C, humidified atmosphere, 5% CO_2_, and 5% or 20% O_2_. The cell culture medium, DMEM, was supplemented with 10% FCS, 1% penicillin/streptomycin, 1% L-glutamine (medium and supplements from Sigma-Aldrich, USA), and 20 mM HEPES (Gibco™, USA). Prior to incubation at 5% O_2_, the cell culture medium was purged with medicinal N_2_, in order to replace the oxygen dissolved in the medium. The decrease of O_2_ level was confirmed with Blood Gas Analyzer Radiometer ABL800 Flex (Radiometer, Denmark). Samples for all measurements described below were taken at day 0 or after 4 days incubation at 5% or 20% O_2_ without additional passaging. Therefore, all cells were measured directly in passage 0 or after detachment of passage 0.

### 2.4. Measurement of Mitochondrial Activity

Mitochondrial respiration of isolated hAMSCs was measured with high-resolution respirometer (Oxygraph-2k, Oroboros Instruments, Austria). At day 0, freshly isolated hAMSCs were seeded with a density of 20,000–30,000 cells/cm^2^ and incubated at oxygen tensions of 5% or 20%. At day 4, the cells were detached and counted. For measurement of ROUTINE respiration, 4 × 10^6^ hAMSCs were resuspended in DMEM at pH 7.2 and 37°C. For measurement of LEAK respiration, ATP synthase was inhibited with 1 *μ*M oligomycin (Sigma-Aldrich, USA). Maximum electron transfer system capacity was measured by titration of carbonyl cyanide-4-(trifluoromethoxy)phenylhydrazone (FCCP, Sigma-Aldrich, USA) in steps of 0.5 *μ*M. Phosphorylation-related respiration was calculated by subtraction of LEAK respiration from ROUTINE respiration (Supplemental Figure 1). Analysis of the data was performed by calculating the slopes of the oxygen concentration curves with Microsoft Excel (Version 14.0.7190.5000 (32 bit)). Sample numbers (biological replicates) *n* = 5–7.

### 2.5. Measurement of Lactate Concentrations

Lactate concentrations were quantified in the cell culture supernatants of 100,000 cells/mL after 4-day incubation of reflected and placental hAMSCs with Blood Gas Analyzer Radiometer ABL800 Flex (Radiometer, Denmark). Sample numbers (biological replicates) *n* = 4.

### 2.6. Adenosine Triphosphate (ATP) Measurement

The samples for measurement of ATP were taken either from freshly isolated hAMSCs or from hAMSCs after cultivation for 4 days at 5% or 20% oxygen. 100,000 cells were pelleted, snap frozen in liquid nitrogen, and stored at −80°C. The cells were homogenized in Precellys tubes with ceramic beads (Keramik-Kit 1.4 mm Peqlab VWR, USA) in a ball mill (CryoMill MM301, Retsch, Germany) with 500 *μ*L of Tris-HCl Buffer (20 mM Tris, 135 mM KCl, pH 7.4). To 100 *μ*L homogenate, 400 *μ*L of boiling 100 mM Tris/4 mM EDTA Buffer (pH 7.75) was added and incubated for 2 min at 100°C and centrifuged at 1000g for 2 min. ATP was determined by ATP Bioluminescence Assay Kit CLS II (Roche, Switzerland) using luciferase reagent with Luminat LB 9507 (Berthold, Germany). Sample numbers (biological replicates) *n* = 3–5.

### 2.7. Determination of Mitochondrial DNA (mtDNA) Copy Number

Cellular DNA was extracted from a pellet of 10,000 hAMSCs using the Tissue & Cell Genomic DNA Purification Kit in accordance with the manufacturer's protocol (GMbiolab Co., Taiwan). The ratio of mtDNA to nDNA was determined as an estimate for the number of mitochondrial genomes per cell using quantitative PCR assays against the single-copy nuclear gene *MYC* and the gene *MT-ND1* representing the minor arc of the mitochondrial genome [53] (Supplemental Table 1). Sample numbers (biological replicates) *n* = 5. The Cq values measured by quantitative PCR were transformed into copy numbers using digital PCR [53].

### 2.8. Reactive Oxygen Species

Electron paramagnetic resonance (EPR) spectra from frozen samples (100,000 cells) were recorded by Miniscope MS200 EPR spectrometer (Magnettech Ltd., Germany) at −196°C (modulation frequency 100 kHz, microwave frequency 9.429 GHz, microwave power 30 mW, modulation amplitude 5 G) as previously described [53]. Intensities of oxidized cyclic hydroxylamine 1-hydroxy-3-carboxy-2,2,5,5-tetramethylpyrrolidine hydrochloride CP-H (3-CP, Noxygen, Germany) signals were recorded at 3359 ± 200 G and quantified by single integrating the low field peak of the 3-CP signal, as previously described [54]. Sample numbers (biological replicates) *n* = 4.

### 2.9. Nitric Oxide Concentration in the Supernatant

Total nitric oxide (NO) levels in the cell culture supernatants of 100,000 cells/mL were analyzed with Sievers 280i-NO Analyzer (General Electrics) as previously described [55]. Briefly, plasma samples were injected through a septum into the glass vessel, where NO species were converted by VCl_3_ to NO_(g)_. A subsequent chemiluminescent reaction with O_3_ caused photon emission, which was converted and displayed as the voltage signal after detection with photomultiplier. Sample numbers (biological replicates) *n* = 5.

### 2.10. Release of Immunoactive Substances

Interleukin- (IL-) 1*β*, IL-6, IL-10, and hepatocyte growth factor (HGF) concentrations were detected in the cell culture supernatant of 100,000 cells/mL after 4-day incubation of hAMSCs at 5% O_2_ and 20% O_2_ with the immunoassay ProcartaPlex™ Human Basic Kit (Thermo Fisher Scientific, Invitrogen, USA), using antibody-coated magnetic beads (Luminex™). Measurement was performed according to the manufacturers' protocol, and absorption was measured with Bio-Plex^®^ 200 instrument (Bio-Rad Laboratories, USA). Sample numbers (biological replicates) *n* = 6.

### 2.11. Statistical Analysis

Data was analyzed using GraphPad Prism software (GraphPad Software, USA). For analysis of day 0 versus day 4, one-way ANOVA was used, followed by the Bonferroni post hoc test in normally distributed data and Kruskal-Wallis combined with Mann-Whitney test in groups showing a non-Gaussian distribution. The Wilcoxon matched pairs test was used to analyze differences in mtDNA copy numbers. Paired *t*-test was used to analyze differences between amniotic regions (reflected versus placental amnion) and oxygen tensions (5% versus 20% oxygen). In all tests, *n* (sample size) represents the number of biological replicates (donors). Results are presented as mean ± SD for normally distributed data. Copy numbers of cellular mtDNA are presented as scatter dot plots. Level of significance was set at 0.05 and is indicated as ^∗^ *p* < 0.05, ^∗∗^ *p* < 0.01, and ^∗∗∗^ *p* < 0.001.

## 3. Results

### 3.1. Measurement of Mitochondrial Activity and Glycolysis

**Figure 1:**
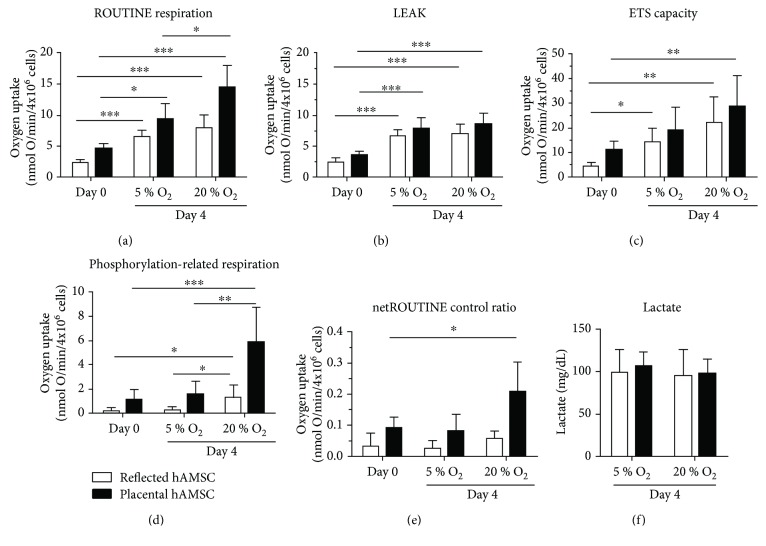
Mitochondrial activity and glycolysis. Mitochondrial respiration was measured in freshly isolated hAMSCs (day 0) and after 4-day incubation at 5% and 20% oxygen (a)–(e). Lactate in the cell culture supernatant was measured at day 4 (5% and 20% oxygen) (f). Cultivation of hAMSCs at 5% and 20% oxygen for 4 days led to elevated ROUTINE respiration (a), LEAK (b), and ETS capacity (c) for both oxygen concentrations but no difference in lactate production (f). Phosphorylation-related respiration was significantly increased only in samples incubated at 20% oxygen (d). This switch to oxidative phosphorylation in hAMSCs incubated at 20% oxygen was confirmed by the netROUTINE control ratio (e) which increases upon stimulation of oxidative phosporylation. *n* = 5–7 (biological replicates), mean ± SD. Abbreviations: ETS: electron transfer system; hAMSCs: human amniotic mesenchymal stromal cells; O_2_: oxygen.

To assess the impact of different oxygen tensions on the quantity of mitochondrial activity, we measured ROUTINE respiration, reflecting the aerobic metabolic activity. Cultivation of hAMSCs at 5% O_2_ and 20% O_2_ for 4 days lead to a significant increase of ROUTINE respiration for both oxygen concentrations. Values were always higher in placental amnion hAMSCs, and the effect was most pronounced in P-hAMSCs incubated at 20% O_2_ (Figure 1(a)).

To determine changes in the quality of mitochondrial activity, we first measured the LEAK state, reflecting proton pumping of the electron transport chain without producing ATP. We observed increased LEAK respiration after 4-day incubation, independent of the oxygen tension and the amniotic region (Figure 1(b)).

Determination of the maximum capacity of the mitochondrial electron transfer system (ETS) showed a similar picture as ROUTINE respiration. The increase in maximum capacity was oxygen-dependent, reaching the highest values in P-hAMSCs incubated at 20% O_2_ (Figure 1(c)).

Calculation of the phosphorylation-related respiration showed a drastic increase in cells incubated at 20% O_2_, and this effect was most pronounced in hAMSCs of placental amnion (Figure 1(d)).

To see which fraction of ETS capacity is utilized to drive the phosphorylation of ADP to ATP, we calculated the “net ROUTINE control ratio” by dividing the phosphorylation-related respiration by the ETS capacity. The strongest increase was observed in P-hAMSCs cultivated at 20% O_2_, indicating that a higher proportion of ETS capacity is utilized to drive ATP synthesis in these cells (Figure 1(e)).

Concentrations of lactate in the cell culture supernatants were measured at day 4, which were neither influenced by the oxygen tension nor the amniotic region (Figure 1(f)).

### 3.2. Measurement of ATP

ATP concentrations in hAMSCs after 4 days in culture increased compared to day 0 with both oxygen concentrations (Supplemental Figure 2).

### 3.3. Counting the Cellular mtDNA Copy Numbers

**Figure 2:**
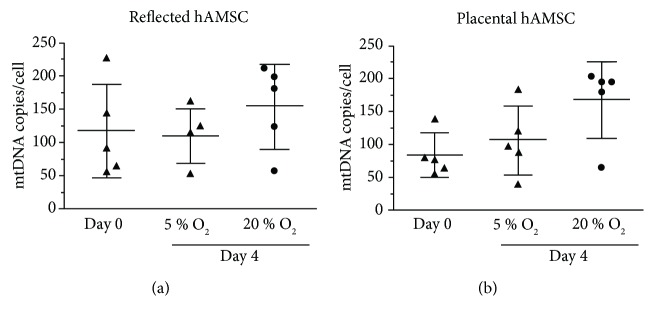
Cellular mitochondrial DNA copy number. The mtDNA copy number per cell was measured by digital PCR in freshly isolated human amniotic mesenchymal stromal cells (day 0) and after 4 days (5% and 20% oxygen) (a) and (b). We observed a trend to an increasing mtDNA copy number in samples incubated at 20% oxygen, but this increase was not significant. *n* = 5 (biological replicates), mean ± SD.

To estimate if the increase in mitochondrial respiration is due to an increase of the cellular mitochondrial content, the mtDNA copies per cell were counted. We observed a trend to an increasing mtDNA copy number in RA-hAMSCs (Figure 2(a)) and P-hAMSC (Figure 2(b)) incubated in 20%, but this increase was not significant.

### 3.4. Generation of Reactive Oxygen and Nitrogen Species

**Figure 3:**
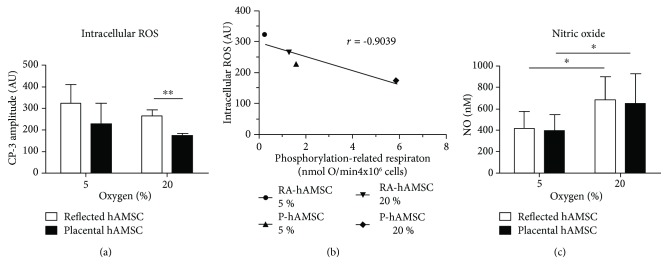
Reactive oxygen and nitrogen species. Intracellular reactive oxygen species levels and nitric oxide levels were measured after 4 days incubation at 5% and 20% oxygen (a)–(c). No significant differences in ROS levels between 5% and 20% oxygen samples were observed (a). hAMSCs from placental amnion showed significantly lower levels of intracellular ROS (a). Calculation of correlation coefficient showed a strong negative association (*r* = −0.9039) between intracellular ROS levels and phosphorylation-related respiration (b). Increased nitric oxide release was detected in cell culture supernatants of samples incubated at 20% oxygen (c). *n* = 5 (biological replicates), mean ± SD. Abbreviations: hAMSCs: human amniotic mesenchymal stromal cells; ROS: reactive oxygen species; RA: reflected amnion; P: placental amnion.

Against our expectations, EPR measurement showed a trend to lower levels of intracellular ROS in hAMSCs incubated at higher oxygen tension. Furthermore, at 20% oxygen, we detected significantly lower levels of intracellular ROS in P-hAMSCs compared to RA-hAMSC (Figure 3(a)). Calculation of correlation between levels of intracellular ROS and phosphorylation-related respiration revealed a strong negative correlation (*r* = −0.9039) between these parameters (Figure 3(b)).

Levels of nitric oxide in the supernatant of the cells showed significant differences between 5% and 20% O_2_. However, within the same oxygen tension, no differences were found between cells of reflected and placental amnions (Figure 3(c)).

### 3.5. Release of Immunoactive Substances

**Figure 4:**
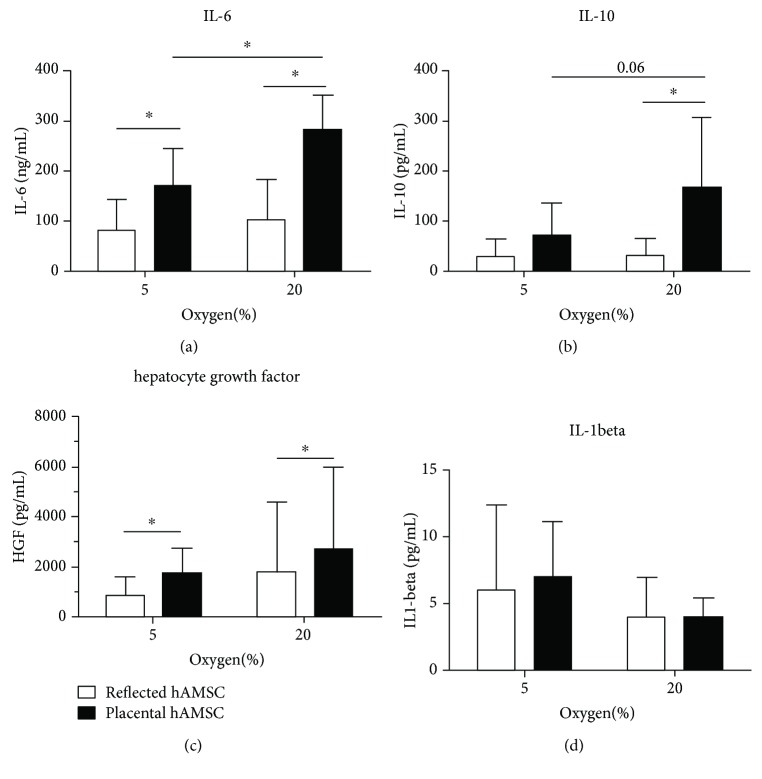
Release of immunoactive substances. Release of immunoactive substances in the cell culture supernatant of hAMSCs of reflected and placental amnion was measured after 4 days (5% and 20% oxygen) incubation (a)–(d). Higher oxygen tension (20%) lead to significantly higher secretion of IL-6 (a) and a trend to higher IL-10 release (b) in hAMSCs from placental amnion and had no effect on the release of hepatocyte growth factor (c) and IL1-beta release (d) compared to lower oxygen tension (5%). Cells from placental amnion released more IL-6 (a), IL-10 (b) and hepatocyte growth factor (c) when compared within cultures at same oxygen concentrations (5% and 20% oxygen). *n* = 6 (biological replicates), mean ± SD. Abbreviations: hAMSCs: human amniotic mesenchymal stromal cells; IL: interleukin.

Measurement of immunoactive substances after 4 days showed significantly higher concentrations of IL-6 in the cell culture supernatant of hAMSCs of placental amnion compared to hAMSCs from reflected amnion (Figure 4(a)). Oxygen tension impacted P-derived hAMSCs to secrete higher levels of IL-6 when incubated at 20% O_2_ compared to 5% O_2_ (Figure 4(a)). A similar pattern was observed when measuring IL-10 release, but significant differences between reflected and placental amnions were only found when cells were incubated at 20% oxygen (Figure 4(b)). Oxygen tension did not impact the release of hepatocyte growth factor (HGF); however, hAMSCs from placental amnion released significantly more HGF compared to hAMSCs from reflected amnion (Figure 4(c)). In cells of both regions, we detected very low concentrations of IL-1beta independent of the applied oxygen tension (Figure 4(d)).

## 4. Discussion

Cultivation of stem cells poses a great challenge, since maintenance of stemness requires niches with very low oxygen tensions. Common cell culture laboratories are usually set up for cultivation at 20% oxygen. Mitochondria are the main oxygen consumers, thereby providing energy via oxidative phosphorylation. Of note, stem cells acquire energy through ATP generation via glycolysis, and the metabolic switch from glycolysis to oxidative phosphorylation changes stem cell fate and cell function (reviewed in [23]).

Many research groups have experimented with bone marrow-derived or adipose tissue-derived mesenchymal stem cells under low or atmospheric oxygen tensions [17, 56–58]. Yet, to our knowledge, the influence of low and high oxygen tensions on hAMSCs under cell culture conditions and especially on their mitochondrial function has not been investigated. Furthermore, as the anti-inflammatory properties of the hAM could be critical in a clinical transplantation situation, we also measured parameters linked to inflammation.

Our data show that the mitochondrial metabolism of freshly isolated hAMSCs in culture is highly sensitive to surrounding oxygen levels. ROUTINE respiration (=LEAK state + phosphorylation-related respiration), reflecting total mitochondrial oxygen consumption, was increased in cultures of reflected amnion-derived hAMSCs (RA-hAMSCs) and placental amnion-derived hAMSCs (P-hAMSCs) in an oxygen-dependent manner. This increase came on one hand from an increase in LEAK state. On the other hand, incubation at 20% oxygen accelerated energy production via oxidative phosphorylation which was demonstrated by phosphorylation-related respiration and the netROUTINE control ratio. Interestingly, this effect was more pronounced in hAMSCs derived from placental amnion. This is in line with previous reports, suggesting the need for low oxygen cell culture conditions, similar to the physiological stem cell niche [59].

The idea to use mitochondrial parameters such as respiratory function as an index for stem cell competence was postulated previously [60, 61]. Our findings confirm that mitochondria-linked considerations should be taken into account in the proceedings of cell therapy in regenerative medicine to ensure quality of therapeutic cells.

We found that the LEAK state, reflecting proton permeability of the inner mitochondrial membrane without producing ATP, increased after 4-day incubation, independent of the oxygen tension and the amniotic region. Such an increase could be caused by different capacities of the electron transfer system (complex I–IV) in different microenvironments. This can be excluded in our study, as stimulation of maximal ETS capacity showed similar increases. Increased ROS production [62] is also unlikely, as we found lower levels of ROS. Interestingly, in stem cells, uncoupling proteins prevent mitochondrial glucose oxidation in response to high substrate concentration and thereby prevent ROS accumulation [63]. Therefore, our results can be explained by the high substrate concentration in common cell culture media compared to *in vivo* conditions. These results indicate that not only nonphysiological oxygen tensions but also nonphysiological substrate concentrations can change the cellular metabolism. We will focus on this matter in future studies.

Lactate concentrations in the supernatants were not influenced by oxygen tension, neither in reflected nor in placental amnion. This is not surprising since 5% oxygen is considered to be the physiological oxygen tension for amniotic cells. In addition, lactate concentrations are more often influenced by other factors than oxygen levels (reviewed in [64]).

Similarly, ATP concentrations are also influenced by many factors. We measured ATP concentrations of freshly isolated hAMSCs, as well as after cultivation at 5% and 20% oxygen tensions for 4 days. We found an increase of ATP concentrations after 4 days of culture, compared to freshly isolated cells. However, the net cellular ATP concentrations represent a steady state between synthesis and consumption. Therefore, ATP concentrations do not reflect actual mitochondrial activity (reviewed in [65, 66]).

We further found a trend to increasing mtDNA copy numbers in P-hAMSCs incubated at 20% oxygen, which is similar to an observation by Chen et al., who found increasing mtDNA copy numbers concomitant with an increasing oxygen consumption rate in the early phase of osteogenic induction [67]. In our study, the data suggest that the higher phosphorylation-related respiration is not due to a higher cellular mitochondrial density but to a higher coupling efficiency between the mitochondrial electron transfer system (complex I–IV) and the ATP synthase.

Surprisingly, although mitochondrial oxidative phosphorylation was increased in hAMSCs incubated at high oxygen tension, we observed a trend to lower levels of intracellular ROS in these cells. Calculation of correlation between phosphorylation-related respiration and intracellular ROS revealed a strong negative correlation between these parameters. Such findings have also been found with bone marrow-derived hMSCs [67]. The authors observed an increased oxygen consumption rate concomitant with decreased intracellular ROS levels. In addition, they detected upregulation of two mitochondrial antioxidant enzymes, manganese superoxide dismutase (Mn-SOD) and catalase [67], and could show that especially Mn-SOD was concurrently upregulated to prevent the accumulation of intracellular ROS.

Intracellular ROS such as superoxide (O_2_^·−^) could also be inactivated by nitric oxide (NO^·^), which, beside its vasodilatory and signalling functions, has also antioxidant effectiveness [68]. In our study, independent of the amniotic region, NO^·^ showed higher levels at 20% oxygen compared to 5% oxygen. This was not surprising, since NO^·^ is predominantly generated in the presence of oxygen [69], except enzyme-independent generation of nitrite occurring in tissues under ischemic conditons [70]. However, in order to develop its full antioxidant capacity, NO^·^ has to be present in excess compared to O_2_^·−^ [71]. In contrast, equimolar concentrations of NO^·^ and O_2_^·−^ or excess of O_2_^·−^ can induce oxidative damage [71]. Thus, modulation of the balance between O_2_^·−^ and NO^·^ can be critical and may impact clinical applications.

For wound healing, increased NO^·^ generation could also be beneficial, as it has been shown to increase angiogenesis and improve neovascularization (reviewed in [72]). However, it can also activate matrix metalloproteinase- (MMP-) 9 (reviewed in [72]), which has been shown to play an important part in the spontaneous rupture of fetal membranes [73], possibly by its extracellular matrix-degrading activity. We suggest taking this fact into consideration while handling or cultivating the hAM under common cell culture conditions at 20% oxygen.

It is also important to note that excessive production of NO^·^ can cause inhibition of mitochondrial respiration, by irreversibly binding to complex I [74] or reversibly binding to complex IV [75]. Such excessive generation of NO^·^ occurs in response to inflammatory stimuli, which cause the upregulation of iNOS via nuclear factor- (NF-) *κ*B or signal transducers and activators of transcription- (STAT-) dependent pathways [76].

Regarding parameters linked to inflammation, high oxygen tension showed the strongest effect on P-derived hAMSCs, which secreted significantly more IL-6, when incubated at 20% oxygen without any additional inflammatory challenge, compared to 5% oxygen. Similar results were found in a recent study with fetal membranes [77], which however, did not analyze reflected and placental amnions separately. IL-6 has been shown to inhibit proliferation of T cells [78] and, interestingly, also human amniotic epithelial cells [79]. Furthermore, Kumar et al. found proinflammatory cytokines in amniotic fluid to induce collagen remodelling, apoptosis, and weakening of cultured human fetal membranes [80]. The differences between the amniotic regions are more pronounced than the influence of the oxygen tension. Placenta-derived hAMSC secreted more IL-6, IL-10, and HGF compared to hAMSC of the reflected region. These data corroborate previous studies, where we and others already showed evidence of regional differences of the hAM [52, 53, 81–85]. In our latter report, we determined for the first time different mtDNA copy numbers in cells of the same type taken from different anatomical regions of the same individual, thus demonstrating that even at a normal physiological state, the cellular mtDNA copy number is tightly controlled not only in a cell type-specific but also in a region-specific manner [53]. Epigenetic regulation of the nuclear and/or mitochondrial genomes represents one of the putative factors that should be studied with regard to these regional alterations of the cellular mtDNA content considering that epigenetic marks are cell-type specific [86, 87] and that demethylation of specific mtDNA sites may cause an elevation of its copy number [88].

We hypothesize that the different anatomical locations of the amniotic regions, one covering the placenta, the other opposite of it, may be the cause for the differences observed. The two different areas may carry out different biological tasks during pregnancy. Furthermore, placental amnion could be supplied with different amounts of oxygen and different patterns of nutrients than reflected amnion. It is, however, not entirely known, how the avascular amniotic membrane is provided with nutrients and oxygen. If oxygen is transferred by diffusion, then, *in utero*, more oxygen might diffuse to placental amnion compared to reflected amnion. This hypothesis is corroborated by the behaviour of mitochondria in human amniotic mesenchymal cells and human amniotic epithelial cells. Compared to reflected amnion, placental amniotic cells show higher phosphorylation-related respiration, a parameter strongly linked to aerobic energy generation [53].

In this study, we found placental amniotic cells to respond to oxygen more readily than reflected amniotic cells. This was seen not only with mitochondrial parameters but also with the release of NO^·^ and IL-6. Similar to NO^·^, IL-6 can also act in a dual role (reviewed in [89]). As recently shown, beside its proinflammatory action, IL-6 can attenuate inflammation [90]. Thus, cultivation under a “nonphysiological” oxygen tension may also have beneficial consequences for cell-based therapy. Hence, we propose that amniotic regions and the influence of oxygen should be taken into account for the cell cultivation for clinical applications of the hAM.

## 5. Conclusion

Taken together, we could show that even short-time cultivation (4 days) at common cell culture conditions (20% oxygen) of freshly isolated hAMSC induced significant changes in mitochondrial function and the release of IL-6. Furthermore, the impact of high oxygen tension can be different, depending on the amniotic region, reflected or placental. Investigation of some selected parameters already showed that cultivation conditions can strongly influence cell physiology.

Mitochondrial activity and immunoactive factors are tightly interconnected with fundamental cellular processes involved in tissue regeneration. These distinctly different properties should be taken into consideration for clinical applications of amniotic cells. Depending on the therapeutic purpose, cultivation conditions of the cells should be chosen carefully for providing the best possible quality of cell therapy.

## Data Availability

The data used to support the findings of this study are available from the corresponding author upon request.

## Abbreviations

ATP: Adenosine triphosphate
CP-H: Cyclic hydroxylamine 1-hydroxy-3-carboxy-2,2,5,5-tetramethylpyrrolidine hydrochloride
EPR: Electron paramagnetic resonance
ETS: Electron transfer system
hAM: Human amniotic membrane
hAMSCs: Human amniotic mesenchymal stromal cells
HGF: Hepatocyte growth factor
IL: Interleukin
iNOS: Inducible nitric oxide synthase
MMP: Matrix metalloproteinase
Mn-SOD: Manganese superoxide dismutase
mtDNA: Mitochondrial DNA
NF: Nuclear factor
P-hAMSCs: Placental amnion-derived hAMSCs
RA-hAMSCs: Reflected amnion-derived hAMSCs
ROS: Reactive oxygen species
STAT: Signal transducers and activators of transcription.

## Supplementary Materials

Supplementary Materials Supplemental Table 1: oligonucleotide sequences of quantitative PCR and digital PCR. Supplemental Figure 1: mitochondrial activity measurement. Representative oxygen consumption trace of placental hAMSCs incubated for 4 days at 20 % oxygen (P, blue), and reflected hAMSCs incubated for 4 days at 5 % oxygen (RA, magenta). 1 = ROUTINE respiration, 2 = LEAK, 3 (D1-2) = phosphorylation-related respiration. Abbreviations: hAMSCs, human amniotic mesenchymal stromal cells. Supplemental Figure 2: adenosine triphosphate concentration (ATP). ATP concentration wasmeasured in 100 000 freshly isolated hAMSCs at day 0, and after 4 days incubation at 5 % and 20 % oxygen. The cells were homogenized and ATP was determined using luciferase reagent with Luminat LB9507. n = 3-5 (biological replicates), mean ± SD, ^∗^ *p* < 0.05 vs day 0. Abbreviations: hAMSCs, humanamniotic mesenchymal stromal cells; O2, oxygen.
