# Supplementary material for: Oxygen Tension Strongly Influences Metabolic Parameters and the Release of Interleukin-6 of Human Amniotic Mesenchymal Stromal Cells In Vitro
Source: Stem Cells Int. 2018 Oct 28;2018:9502451. doi: 10.1155/2018/9502451 (PMC6230389; doi:10.1155/2018/9502451)
Supplement: Supplementary Materials — Supplemental Table 1: oligonucleotide sequences of quantitative PCR and digital PCR. Supplemental Figure 1: mitochondrial activity measurement. Representative oxygen consumption trace of placental hAMSCs incubated for 4 days at 20 % oxygen (P, blue), and reflected hAMSCs incubated for 4 days at 5 % oxygen (RA, magenta). 1 = ROUTINE respiration, 2 = LEAK, 3 (D1-2) = phosphorylation-related respiration. Abbreviations: hAMSCs, human amniotic mesenchymal stromal cells. Supplemental Figure 2: adenosine triphosphate concentration (ATP). ATP concentration wasmeasured in 100 000 freshly isolated hAMSCs at day 0, and after 4 days incubation at 5 % and 20 % oxygen. The cells were homogenized and ATP was determined using luciferase reagent with Luminat LB9507. n = 3-5 (biological replicates), mean ± SD, ∗ p < 0.05 vs day 0. Abbreviations: hAMSCs, humanamniotic mesenchymal stromal cells; O2, oxygen. [file 9502451.f1.pdf]

Supplementary Table 1. Oligonucleotide sequences of quantitative PCR and digital PCR

Gene	Accession number of NCBI	Oligonucleotide sequence (5' to 3')	Size (nucleotides)	Tm (°C)	Length of amplicon (base pairs)	Assay reference (PubMed ID)
MYC	NG_007161.2<br>AY214166.1	F: GCCAGAGGAGGAACGAGCT	19	58	81	11472550
		R: GGGCCTTTTCATTGTTTTCCA	21	60		
		P: FAM-TGCCCTGCGTGACCAGATCC-BHQ1	20	65		
MT-ND1	NC_012920.1	F: CCCTAAAACCCGCCACATC	19	59	68	this study
		R: AGCGATGGTGAGAGCTAAGGTC	22	59		
		P: FAM-CCATCACCC-ZEN-TCTACATCACCGCCC-IBFQ	24	68		

Tm: melting temperature of Primer Express 2.0 software (Applied Biosystems, Foster City, USA) under default settings, F and R: forward and reverse amplification primers, P: sequence of fluorogenic hydrolysis probe, FAM: 6-carboxyfluorescein, BHQ1: Black Hole Quencher 1 (BHQ-1®), ZEN: ZEN™ internal quencher, IBFQ: Iowa Black® fluorescent quencher

Supplementary Figure 1

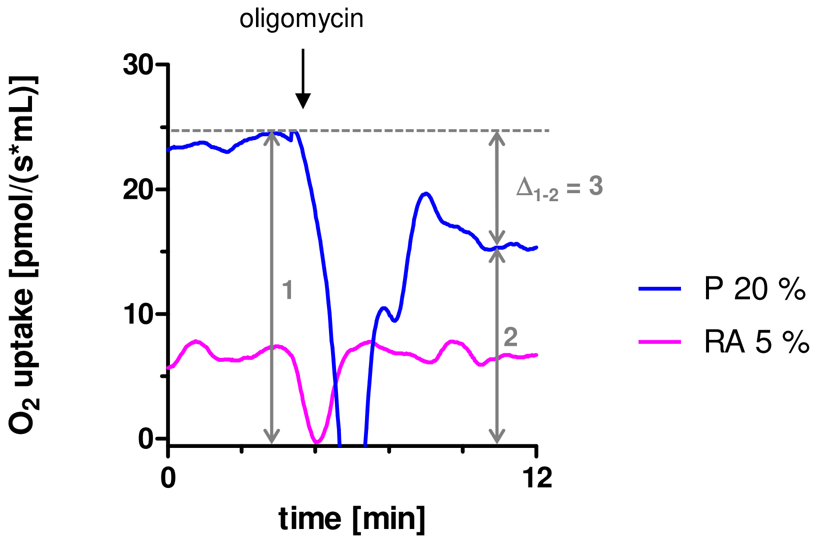

Supplementary Figure 1. Mitochondrial activity measurement. Representative oxygen consumption trace of placental hAMSCs incubated for 4 days at 20 % oxygen (P, blue), and reflected hAMSCs incubated for 4 days at 5 % oxygen (RA, magenta). 1 = ROUTINE respiration, 2 = LEAK, 3 ($\Delta_{1\text{-}2}$) = phosphorylation-related respiration. Abbreviations: hAMSCs, human amniotic mesenchymal stromal cells.

Supplementary Figure 2

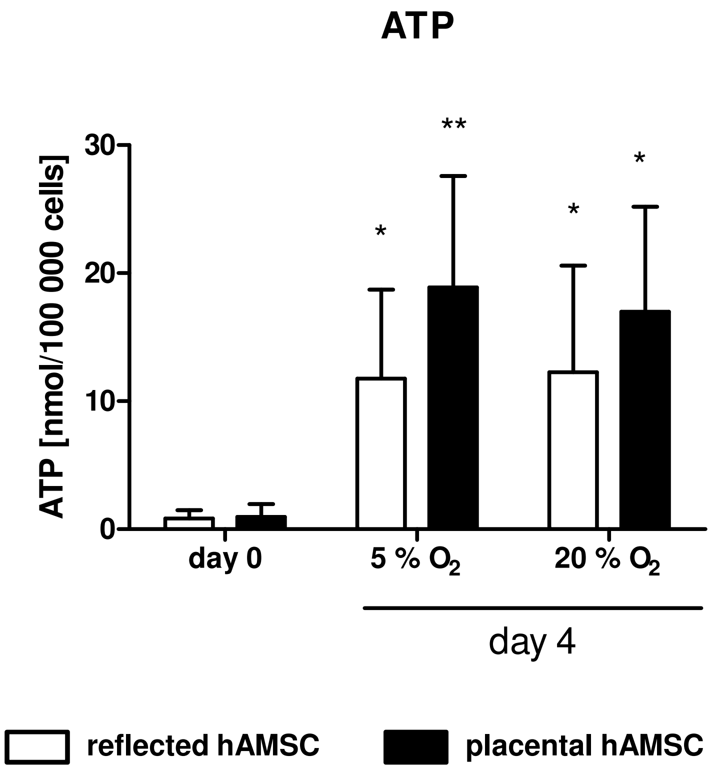

Supplementary Figure 2. Adenosine triphosphate concentration (ATP). ATP concentration was measured in 100 000 freshly isolated hAMSCs at day 0, and after 4 days incubation at 5 % and 20 % oxygen. The cells were homogenized and ATP was determined using luciferase reagent with Luminat LB 9507. n = 3-5 (biological replicates), mean ± SD, *p < 0.05 vs day 0. Abbreviations: hAMSCs, human amniotic mesenchymal stromal cells; O_2, oxygen.
